# Assessment of the reproducibility and accuracy of the Visia^®^ Complexion Analysis Camera System for objective skin analysis of facial wrinkles and skin age

**DOI:** 10.3205/iprs000177

**Published:** 2023-10-02

**Authors:** Helga Henseler

**Affiliations:** 1 Klinik am Rhein, Klinik für Plastische und Ästhetische Chirurgie, Düsseldorf, Germany

**Keywords:** reproducibility, accuracy, precision, wrinkles, facial capture, Visia® Complexion Analysis Camera System, objective measurements, validation

## Abstract

**Objective:**

This study aimed to investigate the reproducibility and accuracy of the Visia^®^ Complexion Analysis Camera System by Canfield Scientific for objective skin analysis.

**Methods:**

Nineteen participants underwent facial capture with the Visia^®^ camera following a standardised protocol. During the first session, the participants sat down and positioned their faces in a capture rig, closed their eyes and had their faces captured from the left, front and right sides, with threefold repetition of the captures from the front side. After 4 weeks, the participants underwent recapture in a similar manner. Based on the frontal views, data for two measurement methods of the Visia^®^ camera system, the absolute scores and the percentiles, were obtained with regard to the skin criterion wrinkles via automated software calculation. Means and standard deviations were evaluated. Based on the side views, the data for the Truskin Ages^®^ were calculated by the Visia^®^ camera system and compared with the calendrical ages, which served as the gold standard for comparison.

**Results:**

In the assessment of the reproducibility of the data of the capture system the standard deviation from the frontal captures among all participants was about 3% when the absolute scores of the wrinkles were compared with each other; specifically, the average deviation was 3.36% during the first capture session and 3.4% during the second capture session. Meanwhile, the standard deviation of the measurements was about 9% when the percentiles were compared; specifically, the average deviation was 8.2% during the first capture session and 10.7% during the second capture session. In the assessment of the accuracy the correlation between the calendrical age and the calculated Truskin Age^®^ for both facial sides was very high at a correlation coefficient rho value of >0.8 (right side: r=0.896; left side: r=0.827) and statistically significant at a p-value of <0.001. The average calendrical age and Truskin Age^®^ deviated only slightly from each other and did not differ significantly (right side: p=0.174; left side: p=0.190). The Truskin Age^®^ was slightly higher than the calendrical age by a mean value of 1.37 years for both facial sides. The analysis of the absolute differences revealed that in 50% of the cases, there was a maximum difference of 3 years, and in 75% of the cases, there were maximum differences of 4.5 years for the right side and 5.5 years for the left side.

**Conclusion:**

The assessment of the reproducibility and accuracy of the objective measurement method, the Visia^®^ camera system, contributed to the validation of the system. The evaluation of the reproducibility revealed a satisfactory precision of the repeated captures when investigating facial wrinkles. Absolute scores should be preferred over percentiles owing to their better precision. The calculation of the accuracy of the Truskin Age^®^ data from the Visia^®^ camera system revealed only a slight deviation from the true calendrical ages. The correlation between both data groups was highly significant.

## Zusammenfassung

**Ziel:**

Die Studie zielte auf die Untersuchung der Reproduzierbarkeit und Genauigkeit der Visia^®^ Kamera der Firma Canfield Scientific bei der objektiven Hautanalyse.

**Methode:**

Neunzehn Teilnehmerinnen unterzogen sich einem standardisierten Protokoll folgend Bildaufnahmen des Gesichtes mittels der Visia^®^ Kamera. Während der ersten Bildaufnahmesitzung nahmen die Teilnehmerinnen Platz und legten ihr Gesicht in ein Bildaufnahmegestell, schlossen die Augen und ihre Gesichter wurden von links, vorne und rechts aufgenommen, mit dreifacher Wiederholung der Bildaufnahmen von vorne. Nach vier Wochen unterzogen sich die Teilnehmerinnen erneuten Bildaufnahmen in gleicher Weise. Basierend auf den frontalen Ansichten wurden die Daten von zwei Messmethoden des Visia^®^ Kamerasystems, die absoluten Werte und Perzentilen, im Hinblick auf das Hautkriterium der Falten durch automatisierte Softwareanalyse ermittelt. Die Mittelwerte und Standardabweichungen wurden evaluiert. Basierend auf den seitlichen Aufnahmen wurden die Daten der Truskin Ages^®^ des Visia^®^ Kamerasystem berechnet und mit dem kalendarischen Alter verglichen, welches als Goldstandard für den Vergleich diente.

**Ergebnisse:**

Bei der Untersuchung der Wiederholbarkeit der Daten des Bildaufnahmesystems betrug die Standardabweichung der Bildaufnahmen von vorne bei allen Teilnehmerinnen ungefähr 3% beim Vergleich der absoluten Werte der Falten untereinander; genau betrug die durchschnittliche Abweichung 3,36% während der ersten Bildaufnahmesitzung und 3,4% während der zweiten Bildaufnahmesitzung. Demgegenüber betrug die Standardabweichung der Messungen ungefähr 9% beim Vergleich der Perzentilen miteinander; genau betrug die durchschnittliche Abweichung 8,2% während der ersten Bildaufnahmesitzung und 10,7% während der zweiten Bildaufnahmesitzung. Bei der Untersuchung der Genauigkeit war die Korrelation zwischen dem kalendarischen Alter und dem kalkulierten Truskin Age^®^ für beide Gesichtshälften sehr hoch bei einem Korrelationskoeffizienten rho von >0.8 (rechte Seite: r=0.896; linke Seite: r=0.827) und war statistisch signifikant bei p<0.001. Das durchschnittliche kalendarische Alter und das Truskin Age^®^ wichen nur leichtgradig voneinander ab und unterschieden sich nicht signifikant (rechte Seite: p=0.174; linke Seite: p=0.190). Das Truskin Age^®^ ergab sich als leichtgradig höher als das kalendarische Alter bei einem Mittelwert von 1,37 Jahren für beide Gesichtshälften. Die Analyse der absoluten Differenzen ergab in 50% der Fälle einen maximalen Unterschied von 3 Jahren und in 75% der Fälle ergaben sich maximale Unterschiede von 4,5 Jahren für die rechte Gesichtshälfte und 5,5 Jahren für die linke Gesichtshälfte.

**Schlussfolgerung:**

Die Untersuchung der Reproduzierbarkeit und Genauigkeit der objektiven Messmethode, des Visia^®^ Kamera Systems, trug zur Validierung des Systems bei. Die Untersuchung der Wiederholbarkeit ergab eine zufriedenstellende Präzision bei den wiederholten Bildaufnahmen bei der Untersuchung der Gesichtsfalten. Die Daten der absoluten Werte sind denen der Perzentilen aufgrund der besseren Präzision vorzuziehen. Die Berechnung der Genauigkeit der Daten des Truskin Age^®^ durch das Visia^®^ Kamerasystem ergab nur eine leichtgradige Abweichung vom wahren kalendarischen Alter. Die Korrelation beider Datengruppen war hochsignifikant.

## Introduction

The validation of an objective measurement method is complex and involves the investigation of the single aspects of the system. Digital imaging for facial capture and analysis has advanced predominantly in the field of maxillofacial surgery in the last three decades [1], [2], [3]. However, concerns regarding the quality of capture systems have emerged. Several studies have thus attempted to validate the performance of imaging technology [4], [5], [6], [7]. What these studies have in common is the assessment of the reproducibility and accuracy of data from capture systems for validation purposes. Part of the examinations is the clarification of how skin surface features and symmetry aspects are displayed.

Recently lip scarring has been examined, and asymmetry assessments have been conducted as well as objective methods in comparison with subjective assessments have been investigated [8]. The importance of validating software packages has been outlined [9]. While software packages are predominantly based on the calculations and analyses of computer scientists, different aspects from the perspective of users arise. During the initial steps of using a new capture system, it is necessary to understand the multiple aspects and their possible applications owing to the complexity of modern software applications. Consequently, there is a certain learning curve [10]. In due course, validation and re-evaluation must be conducted [11].

Apart from maxillofacial surgery, capture systems have been applied in other fields, such as two- and three-dimensional objective assessments of skin conditions [12], [13], [14]. Some studies have applied a modern capture system for the human skin – the Visia^®^ Complexion Analysis Camera System by Canfield Scientific Inc., USA. While an overview of this capture system with its several aspects is of interest [10], a detailed examination of certain individual aspects is needed in due course. This was pursued in the present study.

## Methods

This study was conducted among 19 participants who underwent capture with the Visia^®^ camera system. Facial image capture was conducted following a standardised protocol. During the first session, termed capture 1, the participants positioned their faces in the capture rig, closed their eyes and had their faces captured from the left, front and right sides, with threefold repetition of the captures from the front side. After 4 weeks, the participants underwent recapture in a similar manner (capture 2).

**Figure 1:**
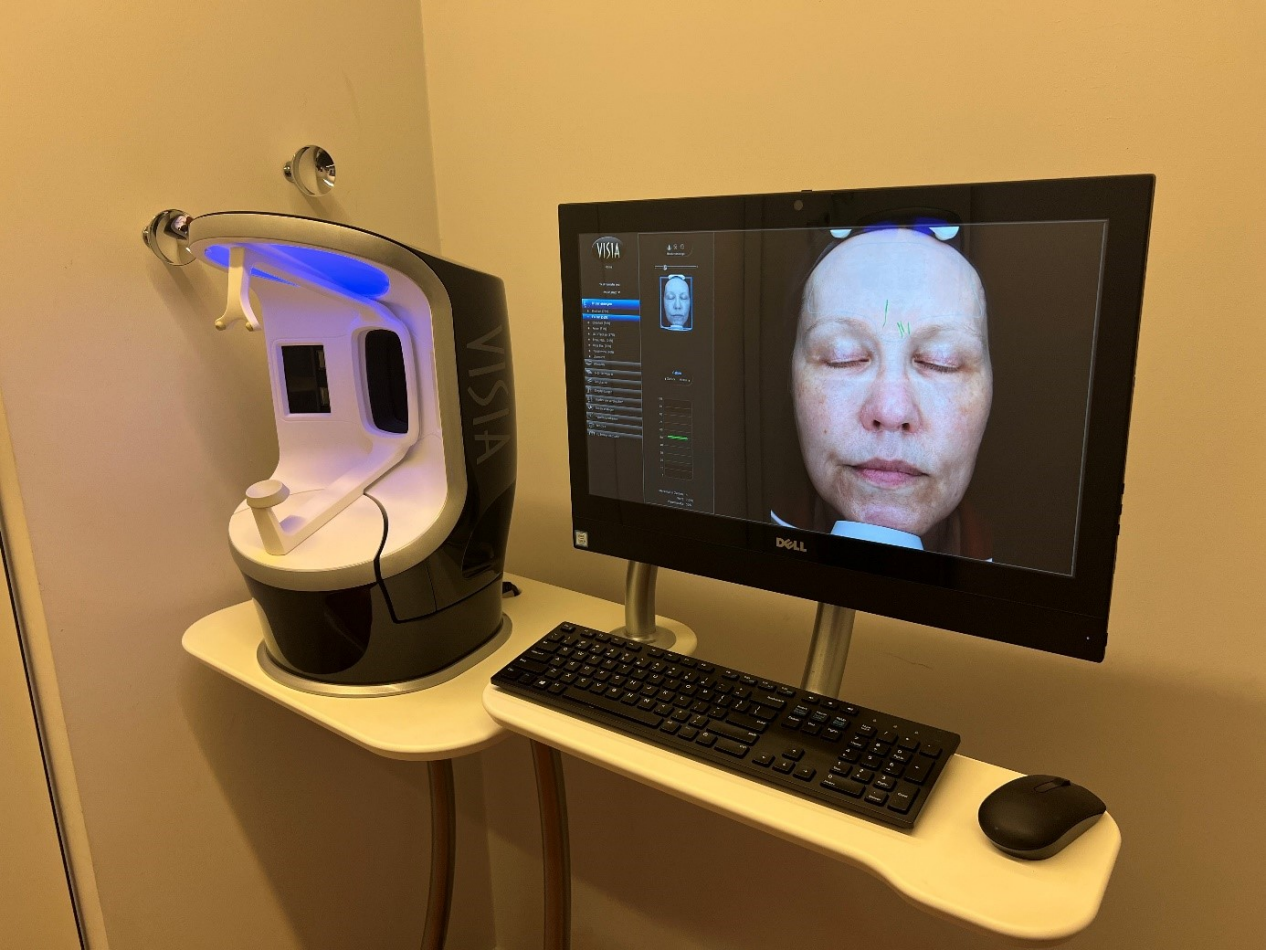
Visia^®^ camera system for the visualisation of several skin aspects

**Figure 2:**
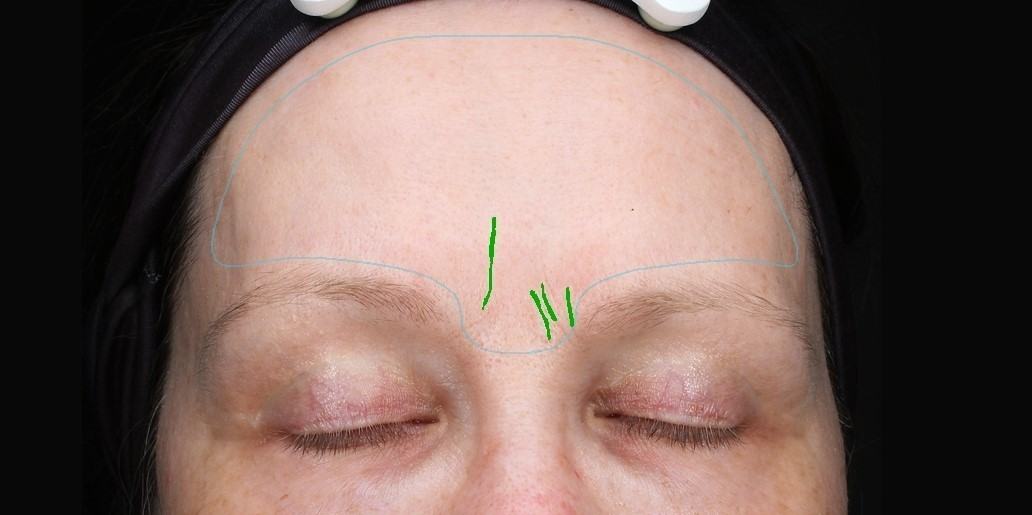
Visualisation of skin wrinkles from the frontal views via software analysis

The images of the Visia^®^ camera system were evaluated on the basis of the detectability of the skin criterion wrinkles from the frontal views via a software analysis (Figure 1 (Fig. 1) and Figure 2 (Fig. 2)).

For the assessment of the reproducibility of the capture system the absolute scores as well as the percentile data for the skin criterion wrinkles were obtained from the three repeated frontal captures. Means and standard deviations were calculated for both measurement methods and both points in time. The standard deviations are a measure for the precision of the capture system.

The measurements were calculated using a spreadsheet calculation programme (LibreOffice Calc). The images were built using the R package ‘ggplot’ (R Core Team 2016) (https://www.R-project.org).

For the assessment of the accuracy of the capture data from the side views, the Truskin Ages^®^ from the Visia^®^ camera system were obtained and compared with the calendrical ages, which served as the gold standard parameters for comparison. As the Visia^®^ allows the calculation of the Truskin Ages^®^ only from the right and left sides of the facial captures, only these views were included in the analysis. 

**Figure 3:**
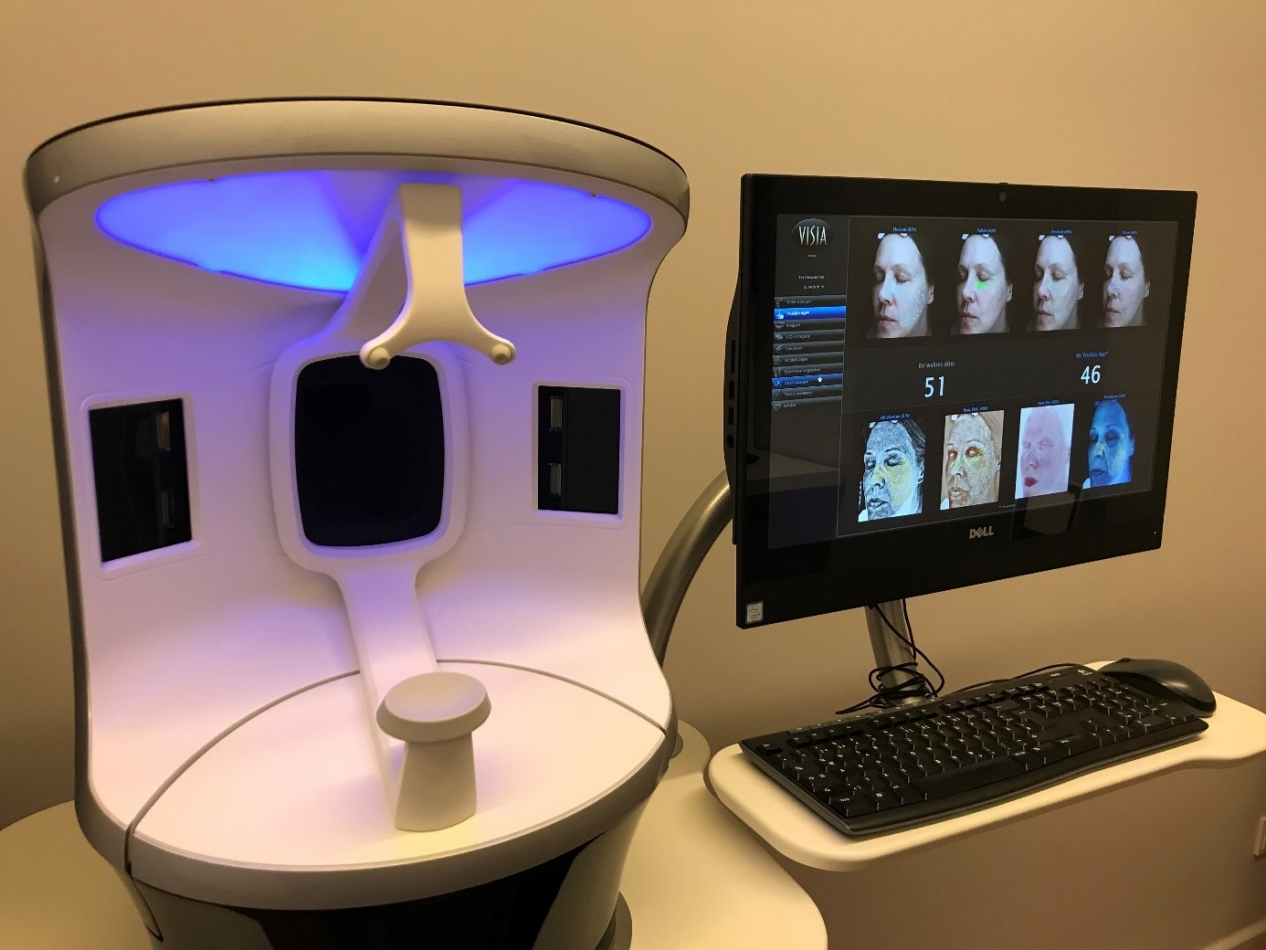
Sample image of the left side of a participant’s face captured using the Visia^®^ camera system, as well as the calendrical true age (51 years) and calculated Truskin Age^®^ (46 years) derived via software analysis

For illustration purposes, Figure 3 (Fig. 3) shows a sample image of a participant captured using the Visia^®^ camera system.

The camera applies several types of flashes to present eight different skin aspects, which are highlighted in different colours. The true calendrical age of the participant (written on the left hand side of the image) as well as the calculated Truskin Age^®^ (written on the right hand side of the image) derived from the data of the eight skin aspects via software analysis are shown.

For the assessment of the accuracy, Truskin Age^®^ data from capture 1 were included in the analysis in order to compare them to the calendrical ages. 

The Spearman correlation coefficient was utilised [15] to evaluate the relationship between the calculated Truskin Age^®^ and the true calendrical age.

For the statistical analysis, the BIAS software [16] was used.

## Results

All participants returned for capture 2. The facial images were captured without any problems in all participants.

### Assessment of reproducibility

The calculated standard deviations from the repeated captures reflected the precision of the capture system in accordance with the skin criterion wrinkles among all participants. The standard deviation revealed the extent to which the measurements differed from each other in accordance with the same criterion. 

The average reproducibility of the data from the captures of all participants was about 3% when the absolute scores of the wrinkles were compared to each other; specifically, the average reproducibility was 3.36% during capture 1 and 3.4% during capture 2. Meanwhile, the average reproducibility of the measurements was about 9% when the percentiles were compared, specifically, 8.2% during capture 1 and 10.7% during capture 2.

The means and standard deviations of the measurements are displayed in Table 1 (Tab. 1).

**Figure 4:**
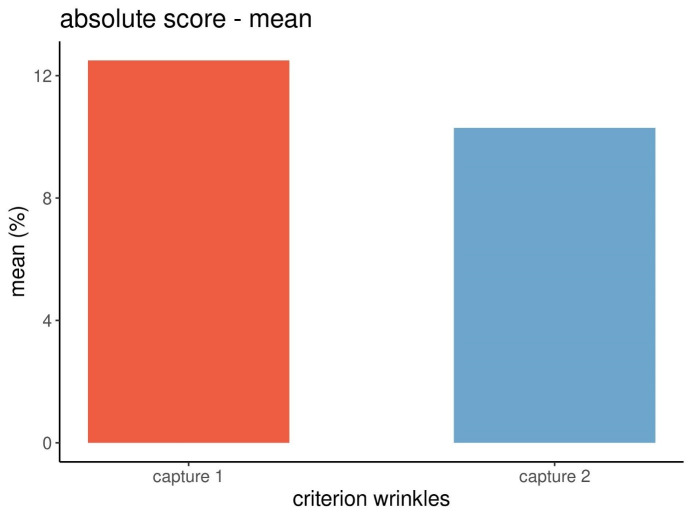
Mean absolute scores of wrinkles from three repeated captures at each point in time

Figure 4 (Fig. 4) displays the means of the calculated absolute scores of the wrinkles.

The measurements were calculated separately for both capture sessions, as only the data during the immediate repetition of the three captures in each capture session surrounding all factors remained the same, which could be different between both points in time.

The differences of the data were further evaluated. The differences of the mean data of the absolute scores of the wrinkles between capture 1 and capture 2 (12.47 and 10.27) were calculated as being not statistically significant according to the Wilcoxon matched pairs test at p=0.376. 

**Figure 5:**
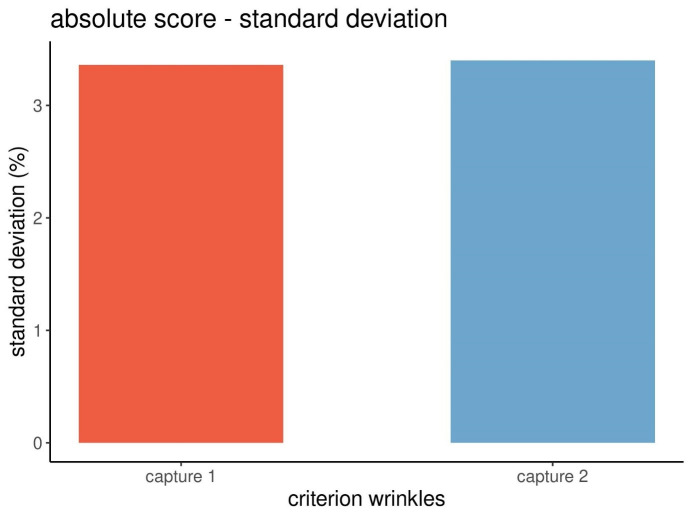
Standard deviations of the absolute scores of wrinkles from three repeated captures at each point in time

Figure 5 (Fig. 5) displays the precision of the measurements based on the standard deviations of the absolute scores of the wrinkles.

As shown in Figure 5 (Fig. 5), the standard deviations of the absolute scores of the wrinkles during captures 1 and 2 differed only slightly (3.36 and 3.40). This finding indicated a good precision of the measurements obtained from the Visia^®^ camera system based on the absolute scores of wrinkles.

**Figure 6:**
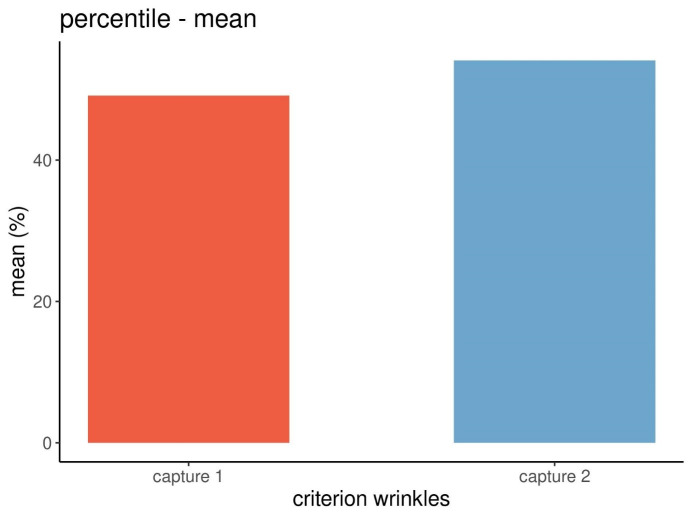
Mean percentiles from three repeated captures at each point in time

Figure 6 (Fig. 6) displays the means of the percentiles of the wrinkles. 

While the mean data of the absolute scores between capture 1 and 2 decreased, albeit not significantly (Figure 4 (Fig. 4)), the mean data for the percentiles increased (Figure 6 (Fig. 6)). This difference is due to the different methods of calculation of the absolute scores and percentiles of the Visia^®^ camera system and was to be expected [11]. 

**Figure 7:**
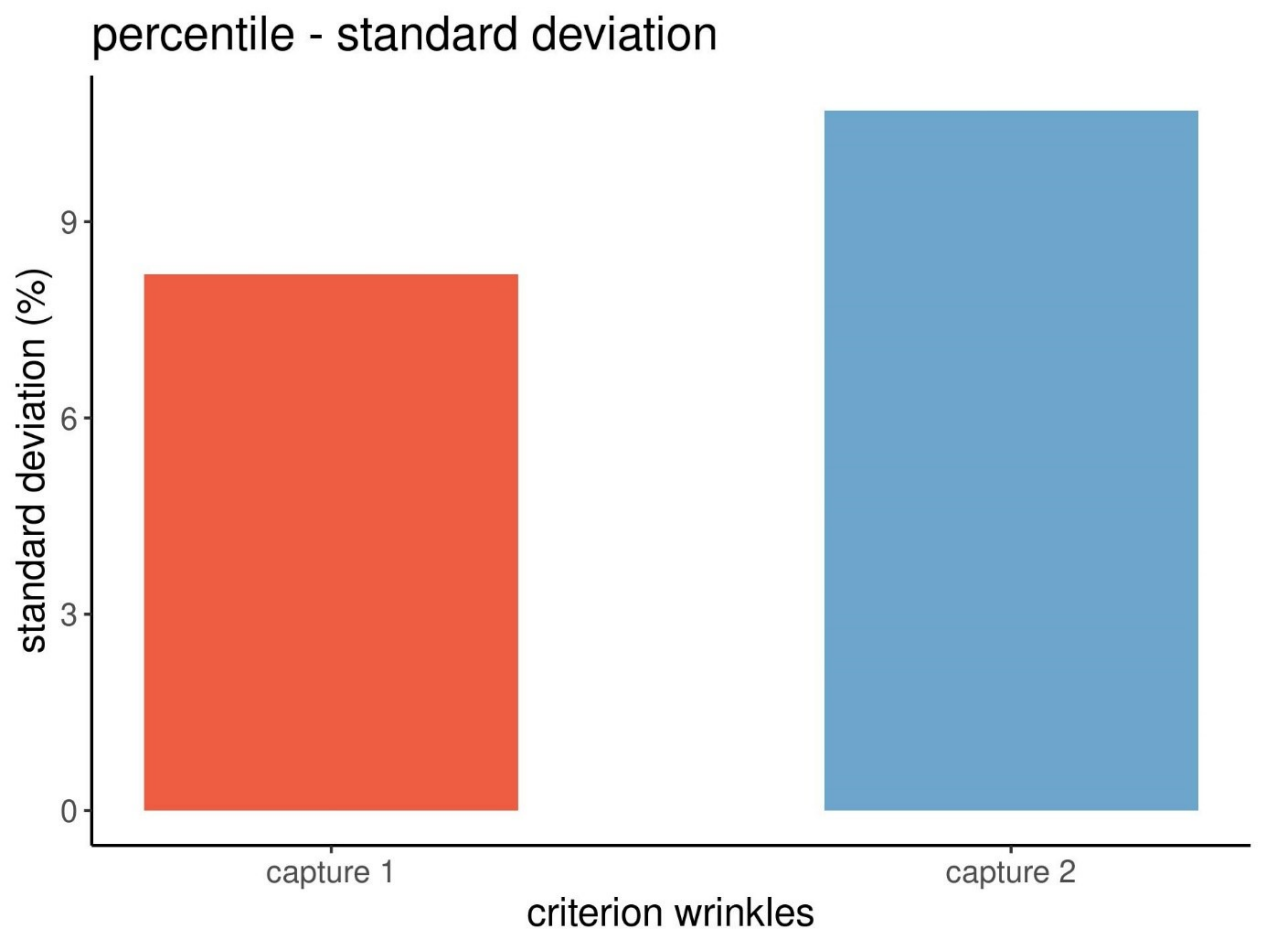
Standard deviations of the percentiles of wrinkles from three repeated captures at each point in time

Figure 7 (Fig. 7) shows the precision of the measurements based on the standard deviations of the percentiles of the wrinkles.

The data and graphical displays revealed that the standard deviations of the percentiles differed more (Figure 7 (Fig. 7)) than those of the absolute scores (Figure 5 (Fig. 5)). Therefore, the precision of the data of the percentiles was less good than the one of the absolute scores. 

### Assessment of accuracy

The accuracy of the data of the true calendrical ages in comparison to the calculated Truskin Ages^®^ during capture 1 was assessed. The Truskin Ages^®^ in both capture sessions differed only minimally (52±9.91 and 52±9.63 years). Therefore, the data of capture session 1 were chosen for the comparison of the calendrical and calculated ages. The data were obtained from the right and left sides of the face, and their general and absolute differences are displayed. Table 2 (Tab. 2) presents the descriptive data of the variables. 

### Correlations

The correlation coefficient between the calendrical age and the calculated Truskin Age^®^ was very high at a rho value of 0.896 for the right side and 0.827 for the left side, which indicated a strong correlation. Further, this correlation was highly significant at a p-value of <0.001. Table 3 (Tab. 3) details the Spearman correlation coefficients.

**Figure 8:**
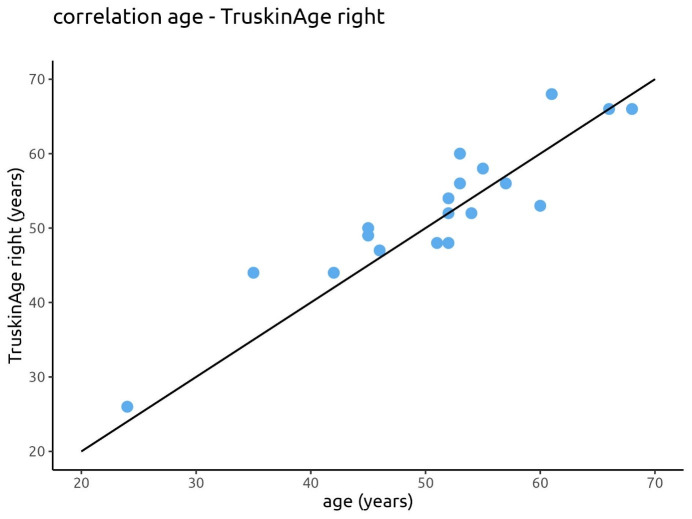
Correlation between the calendrical age and the calculated Truskin Age^®^ for the right facial side

**Figure 9:**
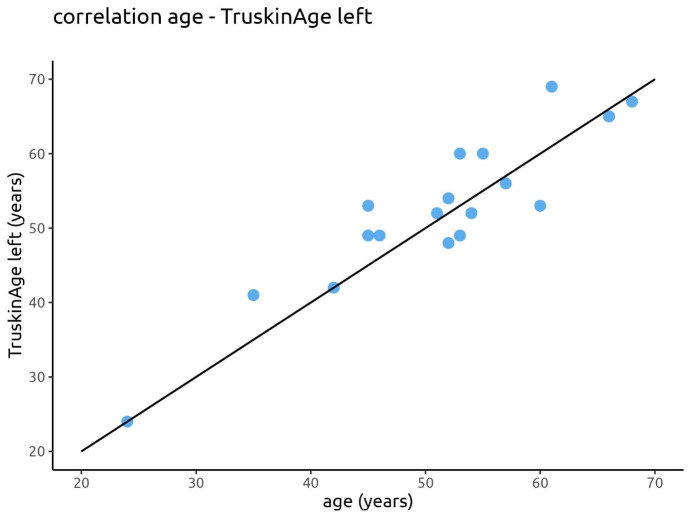
Correlation between the calendrical age and the calculated Truskin Age^®^ for the left facial side

The correlation between the calendrical age and the calculated Truskin Age^®^ for both facial sides is displayed in Figure 8 (Fig. 8) and Figure 9 (Fig. 9).

### Differences

Table 4 (Tab. 4) shows the descriptive data of the calendrical ages and Truskin Ages^®^ for the right and left facial sides. 

The Wilcoxon matched-pairs test conducted for the statistical comparison of the calendrical age with the Truskin Age^®^ for the right and left sides revealed that the calculated Truskin Age^®^ was slightly higher than the calendrical age (52.47 versus 51.11). However, the differences were not significant (p>0.05) at a p-value of 0.174 for the right side and 0.190 for the left side.

### Deviations

Table 5 (Tab. 5) presents the descriptive data of the general differences as well as the absolute differences between the calendrical ages and the Truskin Ages^®^ for both facial sides.

**Figure 10:**
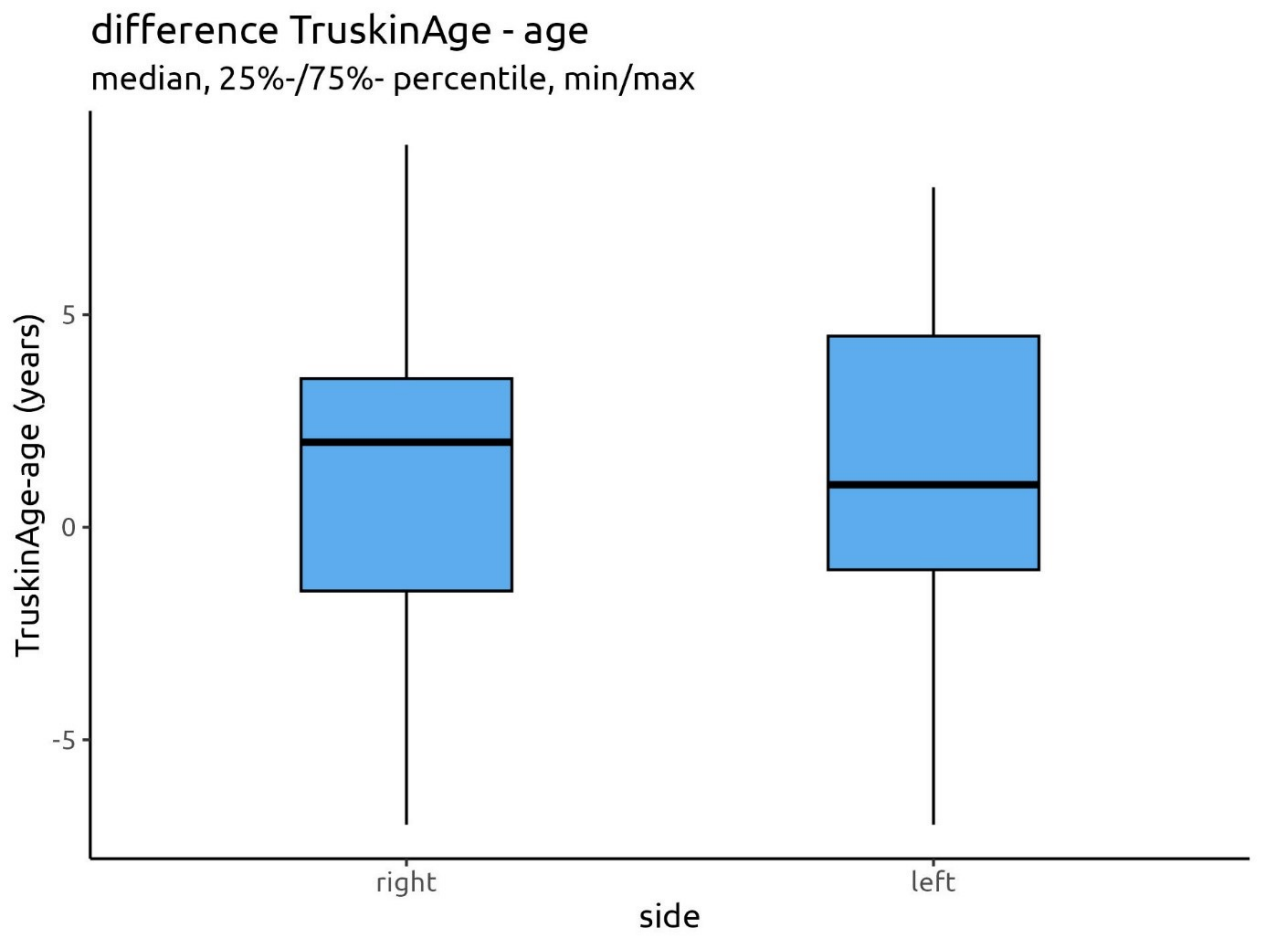
Differences between the calculated Truskin Age^®^ and the calendrical age for the right and left facial sides and their medians and percentiles

The Truskin Ages^®^ were slightly higher than the calendrical ages by an average value of 1.37 years for the right and left sides, with a span of the differences between -7 and +9 years for the right side and -7 and +8 years for the left side. The data analysis revealed a median deviation of 2 years for the right side, with 75% of the cases showing a deviation of less than 3.5 years, and a median deviation of 1 year for the left side, with 75% of the cases showing a deviation of less than 4.5 years (Figure 10 (Fig. 10)).

**Figure 11:**
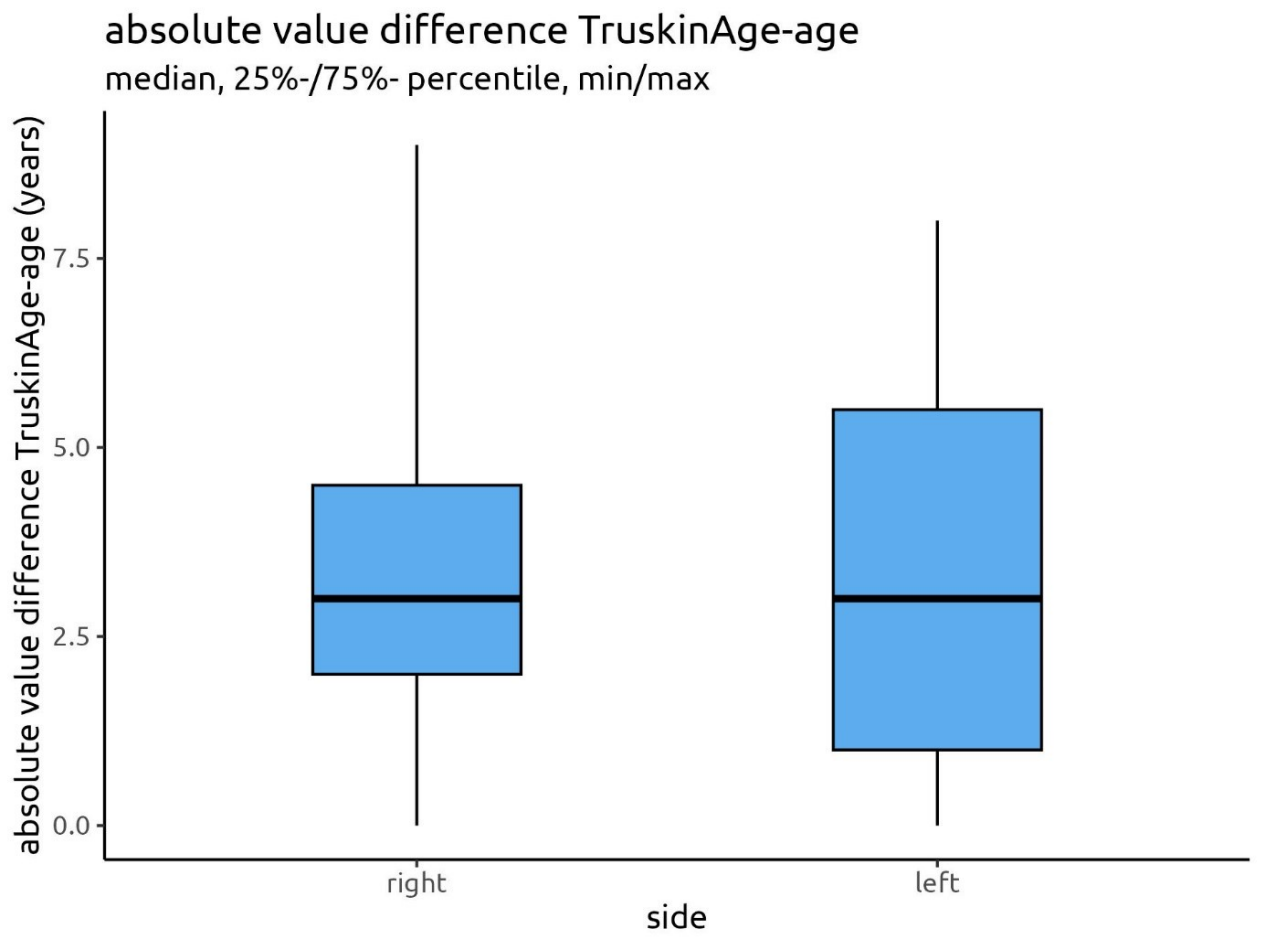
Absolute differences between the calculated Truskin Age^®^ and the calendrical age for the right and left facial sides and their medians and percentiles

The analysis of the absolute values showed a mean deviation of 3.37 and 3.47 years for the right and left facial side, with 50% of the cases showing a deviation of up to 3 years (median) and 75% of the cases showing a deviation of less than 4.5 years for the right side and 5.5 years for the left side (Figure 11 (Fig. 11)).

Figure 10 (Fig. 10) displays the general differences between the Truskin Age^®^ and the calendrical age for both facial sides.

The median of 2.00 for the right facial side and 1.00 for the left facial side and the similar size of the deviations of the data display the small general differences between both data groups, the calculated Truskin Ages^®^ and the calendrical ages. 

Figure 11 (Fig. 11) displays the absolute values of the differences between the Truskin Age^®^ and calendrical age for both facial sides.

The medians of the absolute differences between Truskin Ages^®^ and calendrical ages were 3.00 for both facial sides with some differences in the level of the spread of the data.

## Discussion

The present study investigated the precision of a digital capture system, namely the Visia^®^ complexion analysis camera system by Canfield Scientific Inc., U.S.A. This system yields three different measurement methods: the percentiles, absolute scores and feature counts [11]. Each measurement method has its own purposes, indications, advantages and disadvantages. However, the measurement method that is most prominently presented by the Visia^®^ camera is the one of percentiles. As previously outlined, percentiles reflect the presence of an individual’s skin feature in comparison to a group of people with similar skin characteristics, such as age or skin characteristics according to the scale of Fitzpatrick with consideration of skin colour and ethnicity. Therefore, percentiles allow the comparison of the complexion between an individual and a similar reference group. Percentiles are given as a one- or two-digit figure in percentages; for example, the percentile is 99% if an individual shows no wrinkles at all in contrast to the comparison group. With this measurement, the position within a group of people for comparison and a baseline assessment of the overall skin condition are provided.

As the Visia^®^ camera most prominently presents percentiles, a previous initial investigation with the aim to provide an overview of the system used this measurement method [10]. In comparison, the present study went beyond this previous investigation by expanding the study population as well as the measurement method. Not only percentiles but also absolute scores were examined. Absolute scores offer a measured value in pixels frequently presented as a four- or five-digit figure and display the total size, area and intensity of the skin criterion of interest. These scores are used to investigate the development of a skin feature over time and therefore could aid in answering research questions.

Among the eight different skin criteria commonly assessed by the Visia^®^ camera system one criterion which, together with one other criterion, was previously considered to have the largest variance was selected in this study. This skin criterion was wrinkles. While manufacturers of two- or three-dimensional capture systems are responsible for investigating the technical and mathematical aspects of their products, users are responsible for determining the possible clinical application. This process can be viewed as similar to starting with an initial pilot study to obtain an overview of several features that can be investigated [10], continuing to examine various measurement methods [11] and following with the clinical application [14].

The current study found that the precision of the Visia^®^ camera in assessing wrinkles was rather good based on the absolute scores. However, the data were much less precise when based on the percentiles. This finding supports the previous impression that absolute scores are the preferred measurements in relevant studies. In the present study, the difference in the error was striking, with 3% for the absolute scores compared with 9% for the percentiles. A question of whether this finding would also apply to other skin features arises; however, this would require a new study.

Another aspect of interest would be not only the investigation of the reproducibility of a capture system but also the assessment of the accuracy. As highlighted in the introduction section above, both aspects have been investigated in validation studies of three-dimensional digital imaging systems. While there were hardly any studies on the validation of digital capture systems previously, research efforts have been taken recently, and several study groups have conducted research in this field [17], [18], [19], [20], [21], [22]. However, owing to the large number of different indications, various anatomical areas and two- or three-dimensional digital capture systems for usage, there remains a paucity of relevant studies. For example, no further published clinical studies have evaluated the Visia^®^ camera system as such beyond previous works. Only the application of the camera has been mentioned [23], [24], [25], [26]. Even from the side of biomedical science and engineering the Visia^®^ camera system was not evaluated in its validity as such, but has been used as the reference method for a new proposed model to train deep learning in order to solve the problem of light-tissue interactions [27]. Further, the Visia^®^ camera system was applied as a tool in comparison with other capture systems, but again without an investigation of its own accuracy and reproducibility [28], [29]. Alternatively, the Visia^®^ was used as a reference method in the investigation of rosacea in comparison to physician’s assessment [30].

However, the quality of the capture system in the medical clinical application as such was hardly investigated. While the present study assessed the precision of the objective measurements of wrinkles, the accuracy of the data could not be evaluated. This is because an investigation of the accuracy of a measurement method requires another method that could serve as the true gold standard reference. Such a reference of objective wrinkle measurement is missing in this study as it differs from simply another measurement method for comparison. 

On the contrary, for the calculated Truskin Ages^®^ different outcomes were obtained. As the true calendrical age of the study participants was available, a comparison between this age and the Truskin Age^®^ was possible. Interestingly, a high level of correlation between these ages was found, and the differences were not significant. This comparison of the calculated Truskin Age^®^ with the calendrical age is new. Among the study participants, the calculated age was slightly higher than the calendrical age. However, notably, there was a span of -7 to +8 or 9 years of age difference between the calculated and calendrical ages. Therefore, the individual facial assessments with the Visia^®^ camera system should be interpreted with caution. Over-interpretation of the data must be avoided. This finding is important when using this system in the individual skin analysis of patients or customers. The possible limitations in the interpretation of individual data should be explained. Nevertheless, this study revealed that the Truskin Age^®^ data from the Visia^®^ camera system appeared to present valuable parameters and may thus be used for the evaluation in study groups. Further validation efforts should follow as well as further clinical studies. 

## Conclusion

The assessment of the reproducibility and accuracy of the objective measurement method, the Visia^®^ camera system, contributed to the validation of the system.

The evaluation of the reproducibility revealed a satisfactory precision of the repeated captures when investigating facial wrinkles. Absolute scores should be preferred over percentiles owing to their better precision.

The calculation of the accuracy of the Truskin Age^®^ data from the Visia^®^ camera system revealed only a slight deviation from the true calendrical ages. The correlation between both data groups was highly significant.

## Notes

### Ethical approval

All procedures were performed in accordance with the ethical standards of the institutional and national research committees and with the 1964 Helsinki declaration and its later amendments or comparable ethical standards.

### Competing interests

The author declares that she has no competing interests. The companies were not involved in the conduction of the study or in the analysis or publication of the results.

### Acknowledgements

I thank Dr. Holger Hofheinz and the staff of the Klinik am Rhein for their support in searching for the volunteers and organising the visits of the volunteers to the clinic.

I also thank Dr. Wolfgang Reimers for his statistical advice in the planning of the study and his support in the analysis of the data.

## Figures and Tables

**Table 1:**
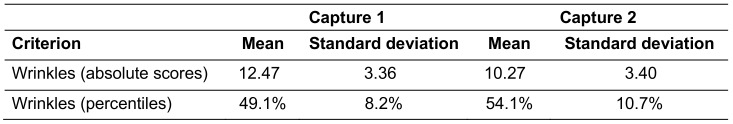
Absolute scores and percentiles of wrinkles

**Table 2:**
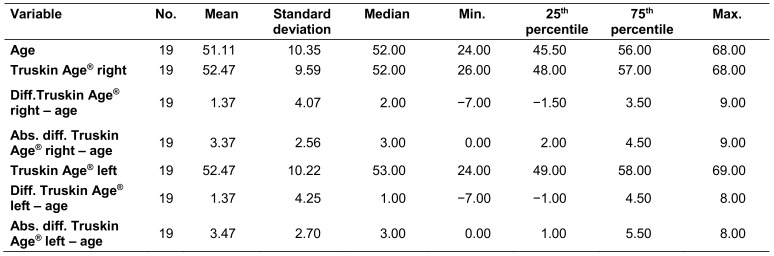
Calendrical ages and calculated Truskin Ages^®^ for both facial sides and their differences

**Table 3:**
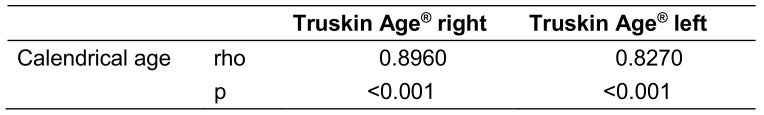
Spearman correlation coefficients

**Table 4:**
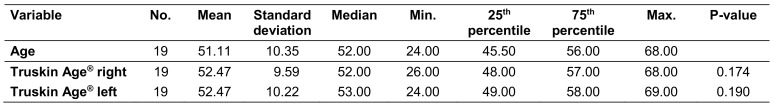
Calendrical ages and Truskin Ages^®^ for both facial sides and their differences

**Table 5:**
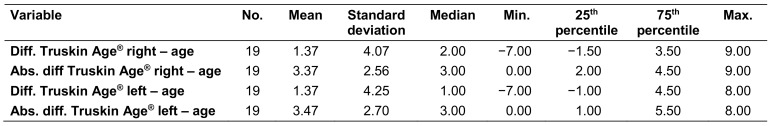
General and absolute differences between the Truskin Ages^®^ and the calendrical ages for both facial sides

## References

[1] Ayoub AF, Wray D, Moos KF, Siebert P, Jin J, Niblett TB, Urquhart C, Mowforth R (1996). Three-dimensional modeling for modern diagnosis and planning in maxillofacial surgery. Int J Adult Orthodon Orthognath Surg.

[2] Hajeer MY, Ayoub AF, Millett DT, Bock M, Siebert JP (2002). Three-dimensional imaging in orthognathic surgery: the clinical application of a new method. Int J Adult Orthodon Orthognath Surg.

[3] Al-Anezi T, Khambay B, Peng MJ, O'Leary E, Ju X, Ayoub A (2013). A new method for automatic tracking of facial landmarks in 3D motion captured images (4D). Int J Oral Maxillofac Surg.

[4] Ghoddousi H, Edler R, Haers P, Wertheim D, Greenhill D (2007). Comparison of three methods of facial measurement. Int J Oral Maxillofac Surg.

[5] Khambay B, Nairn N, Bell A, Miller J, Bowman A, Ayoub AF (2008). Validation and reproducibility of a high-resolution three-dimensional facial imaging system. Br J Oral Maxillofac Surg.

[6] O’Neil M, Khambay B, Bowman A, Moos KF, Barbenel J, Walker F, Ayoub A (2012). Validation of a new method for building a three-dimensional physical model of the skull and dentition. Br J Oral Maxillofac Surg.

[7] Cheung MY, Almukhtar A, Keeling A, Hsung TC, Ju X, McDonald J, Ayoub A, Khambay BS (2016). The Accuracy of Conformation of a Generic Surface Mesh for the Analysis of Facial Soft Tissue Changes. PLoS One.

[8] Ayoub A, Khan A, Aldhanhani A, Alnaser H, Naudi K, Ju X, Gillgrass T, Mossey P (2021). The Validation of an Innovative Method for 3D Capture and Analysis of the Nasolabial Region in Cleft Cases. Cleft Palate Craniofac J.

[9] Shan Z, Hsung RT, Zhang C, Ji J, Choi WS, Wang W, Yang Y, Gu M, Khambay BS (2021). Anthropometric﻿ accuracy of three-dimensional average faces compared to conventional facial measurements. Sci Rep.

[10] Henseler H (2022). Investigation of the precision of the Visia® complexion analysis camera system in the assessment of skin surface features. GMS Interdiscip Plast Reconstr Surg DGPW.

[11] Henseler H (2022). Validation of the Visia® Camera System for skin analysis through assessment of the correlations among the three offered measurements - the percentile, feature count and absolute score - as well as the three capture perspectives, from the left, front and right. GMS Interdiscip Plast Reconstr Surg DGPW.

[12] Sapra S, Stewart JA, Mraud K, Schupp R (2015). A Canadian study of the use of poly-L-lactic acid dermal implant for the treatment of hill and valley acne scarring. Dermatol Surg.

[13] Dissanayake B, Miyamoto K, Purwar A, Chye R, Matsubara A (2019). New image analysis tool for facial pore characterization and assessment. Skin Res Technol.

[14] Henseler H (2022). Measurement of the effects of the IMAGE MD® skin care regimen on skin surface features via modern imaging technology with the Visia® complexion analysis camera system. GMS Interdiscip Plast Reconstr Surg DGPW.

[15] Hedderich J, Sachs J (2020). Angewandte Statistik.

[16] Ackermann H (2022). BiAS für Windows. Programmsystem zur statistischen Datenanalyse, Version 11.12.

[17] Boisnoir A, Decker L, Reine B, Natta F (2007). Validation of an integrated experimental set-up for kinetic and kinematic three-dimensional analyses in a training environment. Sports Biomech.

[18] Skvara H, Burnett P, Jones J, Duschek N, Plassmann P, Thirion JP (2013). Quantification of skin lesions with a 3D stereovision camera system: validation and clinical applications. Skin Res Technol.

[19] Ogawa Y, Wada B, Taniguchi K, Miyasaka S, Imaizumi K (2015). Photo anthropometric variations in Japanese facial features: Establishment of large-sample standard reference data for personal identification using a three-dimensional capture system. Forensic Sci Int.

[20] Jakob V, Küderle A, Kluge F, Klucken J, Eskofier BM, Winkler J, Winterholler M, Gassner H (2021). Validation of a Sensor-Based Gait Analysis System with a Gold-Standard Motion Capture System in Patients with Parkinson's Disease. Sensors (Basel).

[21] Homan K, Yamamoto K, Kadoya K, Ishida N, Iwasaki N (2022). Comprehensive validation of a wearable foot sensor system for estimating spatiotemporal gait parameters by simultaneous three-dimensional optical motion analysis. BMC Sports Sci Med Rehabil.

[22] Li F, Yang Y, Sun X, Qiu Z, Zhang S, Tun TA, Mani B, Nongpiur ME, Chansangpetch S, Ratanawongphaibul K, Manassakorn A, Tantisevi V, Rojanapongpun P, Lin F, Cheng W, Zhou R, Liu Y, Chen Y, Xiong J, Tan M, Aung T, Xu Y, Ting DSW, Zhang X (2022). Digital Gonioscopy Based on Three-dimensional Anterior-Segment OCT: An International Multicenter Study. Ophthalmology.

[23] Villani A, Annunziata MC, Cinelli E, Donnarumma M, Milani M, Fabbrocini G (2020). Efficacy and safety of a new topical gel formulation containing retinol encapsulated in glycospheres and hydroxypinacolone retinoate, an antimicrobial peptide, salicylic acid, glycolic acid and niacinamide for the treatment of mild acne: preliminary results of a 2-month prospective study. G Ital Dermatol Venereol.

[24] Dou W, Yang Q, Yin Y, Fan X, Yang Z, Jian Z, Zhu Y, Wei J, Jing H, Ma X (2021). Fractional microneedle radiofrequency device and fractional erbium-doped glass 1,565-nm device treatment of human facial photoaging: a prospective, split-face, random clinical trial. J Cosmet Laser Ther.

[25] Kim S, Lee J, Park M, Kim H, Kim S, Byun JW, Hwang-Bo J, Park KH (2021). Technique for analyzing the transfer of colored cosmetics onto face masks. Skin Res Technol.

[26] Kobwanthanakun W, Silpa-Archa N, Wongpraparut C, Pruksaekanan C, Manuskiatti W (2021). An evaluation of the course of facial sunscreen coverage and sustainability over an 8-hour workday among outdoor workers. Health Sci Rep.

[27] Jung G, Kim S, Lee J, Yoo S (2023). Deep learning-based optical approach for skin analysis of melanin and hemoglobin distribution. J Biomed Opt.

[28] Linming F, Wei H, Anqi L, Yuanyu C, Heng X, Sushmita P, Yiming L, Li L (2018). Comparison of two skin imaging analysis instruments: The VISIA® from Canfield vs the ANTERA 3D® CS from Miravex. Skin Res Technol.

[29] Wang X, Shu X, Li Z, Huo W, Zou L, Tang Y, Li L (2018). Comparison of two kinds of skin imaging analysis software: VISIA® from Canfield and IPP® from Media Cybernetics. Skin Res Technol.

[30] Pan Y, Jia K, Yan S, Jiang X (2022). Effectiveness of VISIA system in evaluating the severity of rosacea. Skin Res Technol.

